# Missed Jeffery Type II Injury: Treatment in Two Cases and Literature Review

**DOI:** 10.1155/cro/2730280

**Published:** 2026-04-16

**Authors:** Julián Carlos Segura-Nuez, Diana Elena Mandu, Victoria Eugenia Gómez-Palacio, Isabel Parada-Avendaño, Jorge Gil-Albarova

**Affiliations:** ^1^ Orthopaedic Surgery and Traumatology Department, Miguel Servet University Hospital, Zaragoza, Spain, sectorzaragozados.salud.aragon.es; ^2^ Department of Surgery, Faculty of Medicine, University of Zaragoza, Zaragoza, Spain, unizar.es

**Keywords:** children, delayed diagnosis, elbow dislocation, Jeffery Type 2 injury, radial neck fracture

## Abstract

The diagnosis and management of Type 2 Jeffery injuries, particularly when missed in the acute phase, present significant challenges. There are few documented cases of Jeffery fractures in the literature, and limited evidence regarding outcomes following treatment of delayed or missed injuries. We present two cases of undiagnosed Jeffrey Type 2 lesions. Both patients, aged 10 and 8 years, initially presented with elbow pain after falls. The injuries were unrecognised at initial assessment, but were identified at follow‐up 3–4 weeks later. Surgery was performed promptly after diagnosis. In both cases, the postoperative results included full flexion‐extension of the elbow, but residual limitations in supination and pronation were observed. Long‐term follow‐up showed gradual improvement in range of motion, although mild deficits persisted.

In the context of acute elbow dislocation in childhood, the recognition of concomitant Jeffery′s injury is of paramount importance given the potential for significant morbidity and the need for prompt intervention. Open reduction with soft tissue reconstruction and osteosynthesis of the fracture can be an effective treatment, providing satisfactory long‐term clinical and radiographic results, even in cases where the fracture was initially missed. Systematic radiographic assessment of paediatric elbow injuries is therefore essential to avoid misdiagnosis and ensure optimal treatment.

## 1. Introduction

Radial neck fractures represent between 5% and 10% of elbow fractures in children. These fractures can be classified according to the degree of radial head displacement (Judet classification), as well as according to the mechanism of injury.

In 1950, Jeffery proposed a classification of these fractures into two groups. Group 1 comprised cases exhibiting lateral tilting of the radial head as a consequence of valgus stress. In Group 2, the radial head had rotated by 90° in a backward and proximal direction, occupying the posterior part of the elbow joint. Jeffery postulated that this phenomenon was associated with the reduction of a previous posterior dislocation or subluxation of the elbow joint [1]. This is now recognised as Jeffery Type 2 injury. Newman proposed a third mechanism whereby the fracture occurs during posterior elbow dislocation, resulting in anterior displacement of the radial head [2].

These fractures present both diagnostic and therapeutic challenges. Acute cases of Jeffery Type 2 injuries are rarely reported, and the selection of the most appropriate treatment is a source of controversy. Although some published cases report favourable outcomes, particularly in acute or early‐treated injuries, there is limited evidence regarding delayed diagnosis and management of these lesions. This paper presents two cases of Jeffery Type 2 injuries that were unnoticed for several weeks and subsequently treated at this hospital.

## 2. Case 1

**Figure 1:**
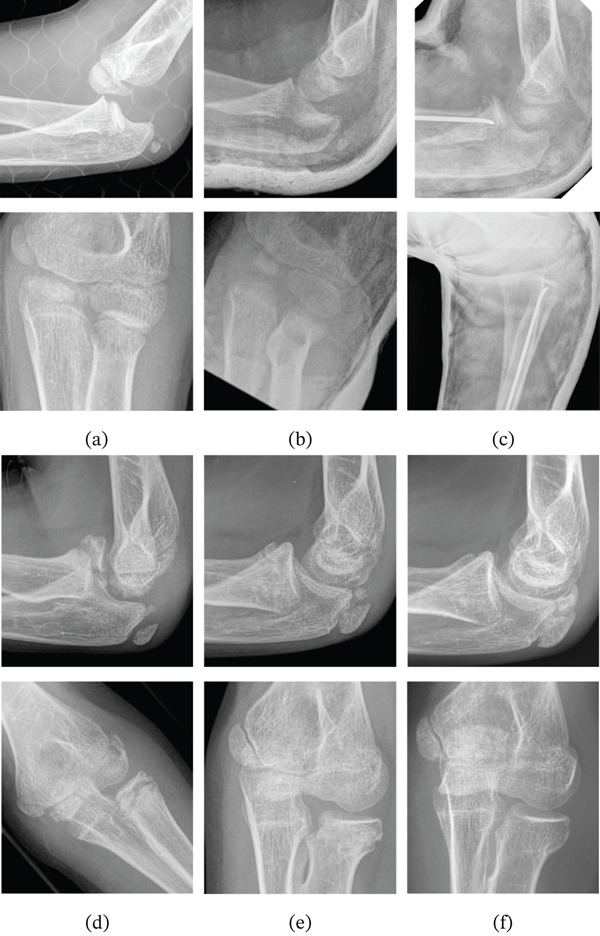
Radiographic evolution of Case 1 (lateral views in the upper row and anteroposterior views in the lower row): (a) dislocation of the left elbow, (b) Jeffery lesion after reduction, (c) open reduction and osteosynthesis with elastic nail, (d) removal of osteosynthesis material at 13 months, (e) follow‐up at 2 years and (f) follow‐up at 4 years.

A 10‐year‐old male patient sustained a posterior dislocation of the right elbow following a fall from a height. Additionally, the initial radiograph demonstrated the presence of a fracture line at the radial neck (Figure 1). Subsequently, a closed reduction of the dislocation was performed, followed by immobilisation with a brachio‐palmar cast. The subsequent radiographic examination, conducted following the reduction of the dislocation, revealed a radial neck fracture with proximal and posterior displacement, as well as 90° angulation of the radial head. These findings were consistent with a Jeffery Type 2 injury. Nevertheless, this fracture was not identified until the 4‐week follow‐up. The patient underwent surgical intervention on the same day.

A lateral Kocher approach was performed. Intraoperative findings included soft tissue interposition and partial resorption of the fracture edges, which made reduction more challenging. After careful debridement, reduction was achieved and fixation was performed using a retrograde elastic intramedullary nail according to the Metaizeau technique [3]. The annular ligament was repaired.

**Figure 2:**
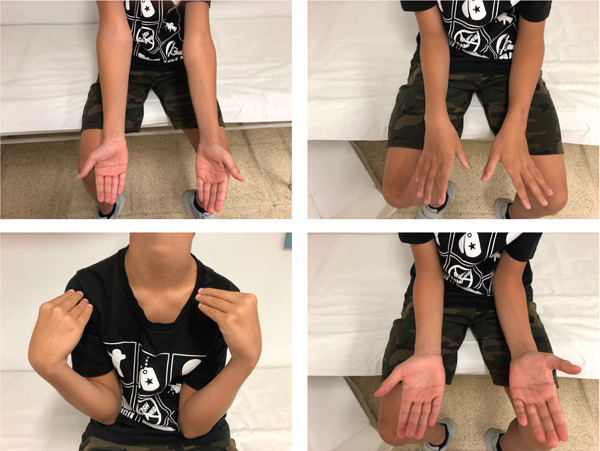
Clinical assessment of Case 1 at 20 months postsurgery.

The arm was immobilised with a cast for 4 weeks, after which mobilisation and rehabilitation were initiated. The nail was removed 13 months after the operation. At 12 months, the patient exhibited full elbow flexion‐extension, though with limited supination (−50°) and pronation (−10°). At 20 months, pronation was complete, yet a supination deficit persisted (−30°) (Figure 2). At final follow‐up, functional outcome was retrospectively assessed using the Mayo Elbow Performance Score (MEPS), with a score of 100, indicating an excellent result. Radiographic and clinical follow‐up demonstrated stable and congruent consolidation and remodelling of the radial head (Figure 1).

## 3. Case 2

**Figure 3:**
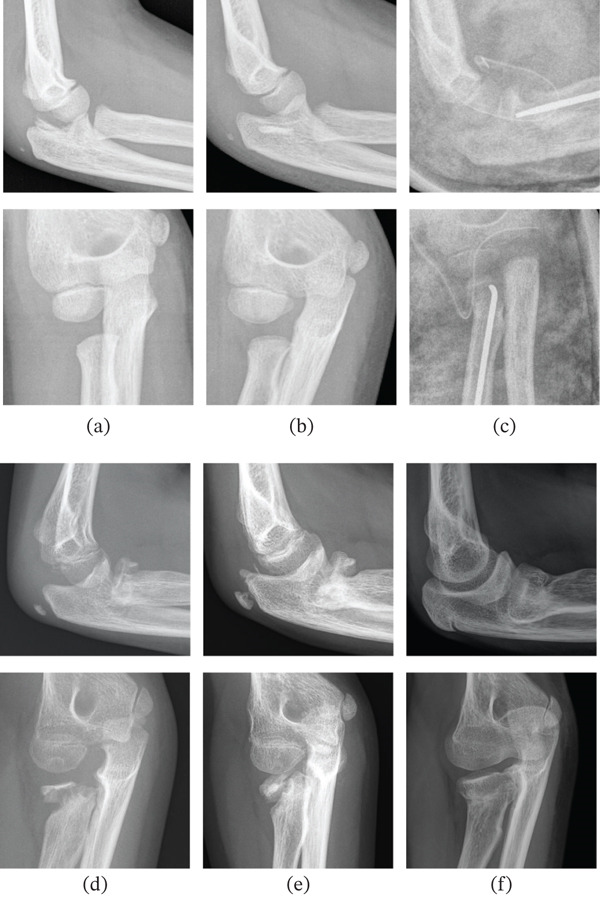
Radiographic evolution of Case 2 (lateral views in the upper row and anteroposterior views in the lower row): (a) acute Jeffery lesion of the right elbow, (b) x‐ray 3 weeks postinjury, (c) open reduction and osteosynthesis with elastic nail, (d) removal of osteosynthesis material at 8 months, (e) Follow‐up at 2 years and (f) follow‐up at 3 years.

An 8‐year‐old female presented with a history of pain and functional impairment in the left elbow following a fall from a height. The initial radiographic examination revealed a radial neck fracture consistent with a Jeffery Type 2 injury, with no additional findings (Figure 3). Although the fracture was not initially diagnosed, a provisional diagnosis of suspected self‐reduced elbow subluxation was established, and the elbow was immobilised for a period of 3 weeks until a follow‐up consultation. At that juncture, a new x‐ray was conducted, which corroborated the initial diagnosis and revealed evidence of bone resorption at the fracture margins.

The following day, the patient underwent open reduction using the Kocher approach. Intraoperatively, fibrous tissue interposition and capsular adhesions were identified and carefully released. The radial head was reduced and stabilised using a retrograde elastic intramedullary nail under fluoroscopic guidance. The annular ligament was repaired. The arm was immobilised in a cast for 4 weeks.

**Figure 4:**
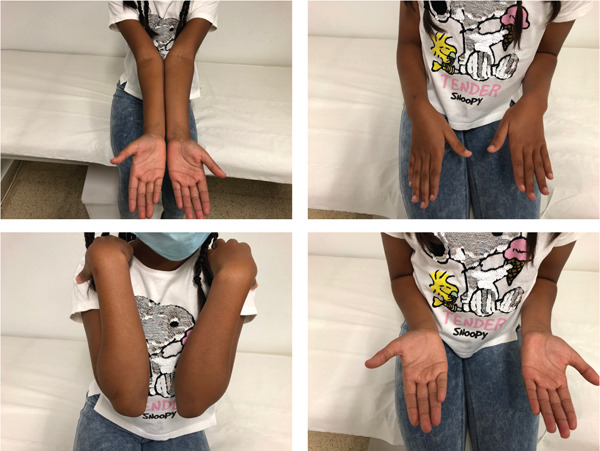
Clinical assessment of Case 2 at 18 months postsurgery.

Thereafter, rehabilitation commenced. Eight months later, the nail was removed, and a follow‐up radiograph demonstrated an anterolateral bony defect of the radial neck. The patient exhibited full flexion‐extension of the elbow, yet limited supination (−10°) and pronation (−30°). At the 18‐month follow‐up, pronation (−20°) showed improvement (Figure 4). Functional outcome was retrospectively assessed using the MEPS, with a score of 100. After 3 years, bone remodelling revealed a hypertrophic radial head that was congruent with the capitellum (Figure 3).

## 4. Discussion

Diagnosing Jeffery′s injury can be challenging, particularly in children under the age of six, when ossification of the radial head typically commences. Following this age, it is vital to be conversant with the ossification sequence of the various elbow centres.

In addition, these injuries may be overlooked due to subtle radiographic findings, especially after reduction of an elbow dislocation. Radiographically, Jeffery Type II lesions are characterised by posterior displacement and approximately 90° rotation of the radial head, which can be difficult to recognise. In doubtful cases, comparison with the contralateral elbow may be helpful, particularly in less experienced settings. Computed tomography (CT) was not performed in our cases, as the diagnosis was established based on plain radiographs and treatment was not delayed.

In the case of acute injuries, the interposition of the condyle limits the potential for reduction. Consequently, open reduction has historically been the preferred treatment for these fractures [4]. In 2006, Chotel et al. presented two cases in which a percutaneous reduction technique was employed, resulting in favourable clinical and radiographic outcomes. In one of the cases, a full range of motion was achieved, symmetrical with the contralateral limb. In the second case, a symmetrical range of motion was achieved in pronation and supination, but the sagittal range of motion was incomplete (5° of extension deficit and a 10° loss of flexion). This technique is contingent upon the existence of a posterior metaphyseal–epiphyseal periosteal attachment between the two radial fragments [5]. In 2019, Papaioannou et al. reported a successful closed reduction case, achieving a satisfactory clinical and radiographic outcome, with a full range of motion [6]. However, there is a risk of complete reversal of the radial head following external manipulation, leading to an ‘upside‐down’ radial head [4, 7].

In instances where the injury is initially undetected, the subsequent treatment process becomes significantly more challenging. The bone edges of the fracture tend to resorb over time, which results in alterations to the congruence between the two ends. Furthermore, soft tissue changes occur due to fragment displacement and subluxation of the proximal radial fragment. This may increase the necessary immobilisation time, complicating reduction, delaying fracture healing and ultimately resulting in functional limitations of the elbow joint.

Radial head resection could be considered as an alternative to open reduction and internal fixation; however, it often results in axial elbow deformity and secondary wrist disorders, which might result in movement restrictions, pain and the need for additional procedures. Therefore, it would be proposed as a salvage treatment in selected patients with a painful, symptomatic elbow joint and a significantly limited range of motion after attempting osteosynthesis [8].

Radial head arthroplasty has also been proposed as an alternative for managing the sequelae of a fracture. Although it appears to improve pain and elbow stability, long‐term outcomes remain uncertain, potentially leading to osteoarthritis or necessitating revision surgeries [9]. In paediatric patients, preservation of the radial head is generally recommended whenever possible due to the risk of long‐term complications associated with resection or prosthetic replacement.

A review of the literature revealed a single published case of chronic Jeffery injury. In this case, Chotel et al. reported a lesion that had remained undetected for 9 years, resulting in periosteal ossification between the radial head and neck. This led to what they termed ‘olecranization’ of the proximal radius and caused significant mobility limitations. The patient presented with a fixed flexion deformity of 70° and an additional flexion range up to 110°. The forearm was locked in 10° of pronation [10].

In our two patients, the lesions remained undetected for 4 and 3 weeks, respectively. Open reduction was therefore chosen as the initial treatment. In both cases, an elastic intramedullary nail was inserted from the distal radial metaphysis to increase stability. For acute injuries, some authors have used K‐wires, absorbable sutures or even simple immobilisation with a cast [4, 6, 11].

Alternative fixation methods include percutaneous techniques and Kirschner wire fixation. Whilst K‐wire fixation is technically simple, it may provide less stable fixation and may increase the risk of physeal injury. Elastic intramedullary nailing offers stable fixation with minimal soft tissue disruption and facilitates early mobilisation.

The results vary according to the published cases, with pronation and supination restrictions being the most common [4]. The clinical and radiographic results of the two cases presented here are favourable despite the delay in diagnosis and treatment. Both patients achieved full flexion‐extension of the elbow, although one had a deficit in supination and the other a limitation in pronation. The MEPS demonstrated excellent results in both cases. We believe that surgical treatment of unrecognised Type 2 Jeffery injuries can be effective even in cases where the fracture is not recognised in the acute phase and is diagnosed within the first month of evolution.

The limitations of this study include the fact that we were only able to identify two cases of Jeffery Type 2 injuries that were initially overlooked. Moreover, the published literature on these injuries is limited, making it challenging to compare results. Regarding chronic injuries, we found only one published case.

## 5. Conclusion

Jeffery′s injury should be suspected in children with elbow dislocation. Open reduction with soft tissue reconstruction and osteosynthesis of the fracture can be an effective treatment with satisfactory long‐term clinical and radiographic results, even in cases where the fracture was not initially detected. A thorough history, physical examination, and systematic radiographic assessment of the painful paediatric elbow are essential.

## Author Contributions

Julián Carlos Segura‐Nuez: conceptualization, data curation, investigation, methodology, writing—original draft. Diana Elena Mandu: data curation, investigation, writing—original draft. Victoria Eugenia Gómez‐Palacio: supervision, writing—review and editing. Isabel Parada‐Avendaño: supervision, writing—review and editing. Jorge Gil‐Albarova: conceptualization, data curation, investigation, methodology, writing—review and editing, supervision.

## Funding

No funding was received for this manuscript.

## Consent

Written informed consent was obtained from the patients′ parents/legal guardians for publication of these case reports and accompanying images.

## Conflicts of Interest

The authors declare no conflicts of interest.

## Data Availability

The data supporting the findings of this study are available from the corresponding author upon reasonable request.
